# Four types of change and self‐other agreement on change in personality traits during college years: A multi‐informant longitudinal study

**DOI:** 10.1111/jopy.12740

**Published:** 2022-07-11

**Authors:** Phuong Linh L. Nguyen, Moin Syed, Colin G. DeYoung

**Affiliations:** ^1^ Department of Psychology University of Minnesota Minneapolis Minnesota USA

**Keywords:** correlated change, individual difference, ipsative profile, mean‐level, multi‐informant, personality development, rank‐order

## Abstract

Research in personality trait change has largely relied on mean‐level and rank‐order change across the lifespan. The current research expanded the literature in several ways: analyzing four types of change and correlated change patterns, obtaining multi‐informant reports, including lower‐order personality traits, and collecting multiple assessments during a short yet important time for college‐attending emerging adults (baseline *N* = 259, *M*_age_ = 18.79). There was little evidence for mean‐level change, yet participants showed significant individual differences such that rank‐ordering and ipsative profiles were much more dynamic than mean score patterns. Informant‐reports from close others demonstrated largely similar patterns: little to no mean‐level change, significant increase in rank‐ordering, and about half of participants reporting configural change mostly in elevation and scatter rather than in profile shapes. Interestingly, there was no correlated change between self and other‐reports. This indicated that close others do not share individuals' perception of their own personality trait change, at least not in the demographic group studied. By examining individual‐level, sample‐level, and multi‐informant perspectives, our thorough investigation provided useful benchmarks for future research to examine the source of variability in change trajectories.

## 1 INTRODUCTION

Personality refers to stable yet dynamic patterns of psychological individual differences, such as traits, characteristic adaptations, and life stories (McAdams & Pals, 2006). The current paper focuses on dispositional personality traits, conceived as “probabilistic descriptions of relatively stable patterns of emotion, motivation, cognition, and behavior, in response to classes of stimuli that have been present in human cultures over evolutionary time” (DeYoung, 2015, p. 35). A large body of research examining trait change across different life stages and in conjunction with important life events suggests some malleability to personality traits. Nonetheless, a majority of existing research focuses on sample‐level mean‐score patterns as obtained through self‐reports of the Big Five domains (Agreeableness, Conscientiousness, Extraversion, Neuroticism, and Openness/Intellect), thus providing only one limited perspective on personality change. The current paper extends the investigation of change to four different types of change and correlated change patterns among a broader set of traits, including lower‐order traits. Our study offers both sample‐level and individual‐level perspectives among our sample of college‐attending emerging adults. We also included longitudinal ratings of personality by other informants to examine the convergence between perceptions of change, if any, between participants and their close others.

By definition, elements of personality must be at least somewhat stable over time. However, much research shows that personality does change. Personality change occurs in different life stages in different forms (Bleidorn & Hopwood, 2018; Schwaba & Bleidorn, 2018), from childhood to middle and older adulthood (Roberts et al., 2006). Change may result due to normal developmental processes and follow established principles such as maturation, role continuity, or cumulative continuity (Donnellan et al., 2007; Roberts et al., 2008). The *maturation principle* posits that people become more agreeable, conscientious, extraverted, and less neurotic with age. At the population level, *cumulative continuity* means there is an increase in rank‐order consistency, and, at the individual level, continuity results partially from consistent roles over time due to *role continuity*. Further, change also occurs due to life events, both with relevant normative events (Bleidorn et al., 2018; Denissen et al., 2019) and with adversity and traumatic or significant events such as psychotherapy, beginning and ending relationships, or natural disasters (Jayawickreme et al., 2020; Rakhshani & Furr, 2020; Roberts et al., 2017; Shiner et al., 2017). Beyond just evidence of change, research has demonstrated the utility of such dynamic conceptualizations of personality and its predictive power for psychological adjustment and psychotherapy progress, physical activity levels, and job performance (Kuppens et al., 2007; Lievens et al., 2018; Mõttus et al., 2017; Nguyen et al., 2020).

The current literature on personality change is extensive, yet several key features are missing or deserve more attention, including different conceptualizations of change beyond mean‐level scores, more fine‐grained personality levels beyond the Big Five domains, informant reports to enhance the well‐documented patterns of self‐reports, and a more intensive short‐term data collection during a period of heightened situational change (e.g., emerging adulthood).

### 1.1 Different types of personality change

Although examining personality change may seem straight‐forward, there are in fact several different approaches and conceptualizations of change that have been unevenly investigated. First and foremost, the functional form of change has been largely limited to the linear *mean‐level change*, in which sample mean scores over time are investigated through multi‐level longitudinal models. For instance, a meta‐analysis of 92 longitudinal studies reported a robust increase in conscientiousness and emotional stability through midlife, which supports the maturation principle of personality development (Roberts et al., 2006). However, there are three other types of change that are investigated to a lesser degree: rank‐order change, ipsative profile change, and individual differences in change (for a review, see: Donnellan & Robins, 2009; Roberts et al., 2012).

*Rank‐order change* is another common approach, which examines the ranking among individuals within the sample over time. Research with large national samples and meta‐analytic techniques has demonstrated an inverted U‐shaped pattern of rank‐order stability, with a peak in late adulthood and a subsequent decrease in old age, which is indicative of the cumulative continuity principle of personality (Lucas & Donnellan, 2011; Roberts & DelVecchio, 2000). However, these studies all relied on cross‐sectional comparisons of test–retest correlations across different age groups, and thus did not strictly demonstrate within‐sample increase in stability over time. Notably, in a cross‐sequential design with four waves spanning roughly three years, Costello and colleagues found no increase in stability during early‐ to middle‐adulthood (2017). In addition, much weaker empirical support for this continuity principle has been demonstrated by decomposing the stable and malleable components of personality in fully longitudinal designs (Trait–State‐Occasion model: Cole et al., 2005; Wagner et al., 2019).

*Individual difference in change* refers to the between‐person variation in change trajectories or slopes over time and has been examined with either the Reliable Change Index (Roberts & Mroczek, 2008) or latent growth curve modeling (Schwaba & Bleidorn, 2018). *Ipsative profile change* is the least investigated approach in the literature, but it provides the most individual‐specific yet holistic picture of change: changes in configurations among different traits are examined by Euclidean distances between individual profiles overtime. Two previous studies reported a high degree of consistency between the beginning and end of college years, with the majority of change relating to elevation (mean‐level using raw scores) or scatter (variability across traits using deviation scores) and much less so in the shape or configuration of personality, assessed through standardized scores of multiple traits (De Fruyt et al., 2006; Robins et al., 2001).

Lastly, examinations of *correlated change* have shown strong correlations among change trajectories of different personality traits (Klimstra et al., 2013). Taken together, these various analyses provide a much more complete picture of change than the common piecemeal approach. The current paper aims to apply the four types of change as well as correlated changes between self and other reports, thus highlighting individual traits, individual subjects, and patterns of interactions and aggregation among traits and subjects over time.

### 1.2 Personality trait levels

The second frequently lacking piece in the current personality change literature is investigation of personality traits at levels other than the Big Five domains (Costa & McCrae, 1985; Goldberg, 1993). Researchers have suggested levels above and below the Big Five domains, such as meta‐traits of Plasticity and Stability (DeYoung, 2006; Digman, 1997), thirty specific facets within the domains (McCrae & Costa, 1992), or the 10 aspects between facets and domains (DeYoung et al., 2007). Fine‐grained lower‐level traits often provide researchers with more substantive information about personality processes and have demonstrated additional predictive power beyond the higher‐order domains (e.g., Dyce & O'Connor, 1998; Reynolds & Clark, 2001; Samuel & Widiger, 2008; Soto & John, 2016). In addition, lower‐order traits often provide more contextual information and thus might be particularly important in the investigation of change over time, as change is more easily observed when we move towards more contextualized and situational constructs, rather than focusing on the universal, decontextualized domains (McAdams, 1994). Although such contextual information likely requires even lower‐ordered constructs, the current study aimed to first extend the investigation to the aspect level and include both the five domains and their 10 lower‐order aspects. Thus, we could further examine whether more specific factors were driving the change at the level of the Big Five, or whether there are divergent patterns of change for the aspects that are obscured at the domain level.

In addition, in comparison with the predominant Big Five domains, there have been limited longitudinal reports of the Big Five Aspect Scale (BFAS: DeYoung et al., 2007), which was designed primarily for the aspect levels with items chosen for their high loadings on only one of the two aspects per domain. Using a four‐wave cross‐sequential design at one‐year intervals that covered a wide age range at baseline (18 to 55 years old), Costello et al. (2017) provided some evidence that personality at the aspect level shows differential mean‐level change patterns when compared to their overarching domains. Beyond the BFAS taxonomy, the notion that change patterns might differ between lower‐level or domain‐level traits has been supported by various research programs including facets within Extraversion (Roberts et al., 2006), Conscientiousness, and Openness (Soto et al., 2012).

### 1.3 Self‐ versus other‐reports

Longitudinal research into personality development has been extensive; nonetheless, much of the literature has relied exclusively on self‐reports. Cross‐sectional results from a large sample of 10,000 individuals suggested that the overall patterns of self‐reported age trajectories in personality traits were reflected in informant‐reports; however, there were discrepancies in the timing and magnitude of such effects (Rohrer et al., 2018). Self‐other agreement in personality traits also increased with age. Although such results provided important initial evidence of age effects, any longitudinal conclusions require longitudinal assessments.

To date, studies with both self‐ and other‐reports of personality across time remain rare, and the pattern of results is inconsistent. A large study (*N* = 1630) of older adults showed strong longitudinal self‐other agreement across the Big Five domains with three waves at approximately 6.5‐year‐intervals (Oltmanns et al., 2020). For emerging and middle adulthood, research has examined different levels of acquaintances across shorter intervals, including new roommates for 15 weeks (Kurtz & Sherker, 2003), newly‐wed couples across 2 years (Watson & Humrichouse, 2006), and romantic partners across 1.5 years (Lenhausen et al., 2021). As could be expected, differences in length and frequency of assessments produced different patterns of results that are difficult to synthesize. Notably, Lenhausen and colleagues' (2021) investigation included four assessment waves across 1.5 years that allowed for a more direct examination of self‐ and other‐perceived change, instead of the change in self‐other agreement as made possible by fewer time points or a more condensed time period. They demonstrated little difference between partners' reports in terms of mean‐level and rank‐order stability, while recognizing the importance of expanding to different dyadic types beyond romantic relationships. Hence, the current paper contributes to fill this gap by including longitudinal multiple‐informant assessments across four time points. To examine the general other‐perception of change, participants were free to elect any informant(s) of their choice, including classmates, close friends, romantic partners, and family members.

### 1.4 Emerging adulthood

Emerging adulthood, which includes ages ranging from 18 to 30 years (Arnett, 2000), is the age group that shows most prominent individual differences in personality trait change (Bleidorn & Schwaba, 2017; Roberts & Davis, 2016; Schwaba & Bleidorn, 2018). Individuals in this demographic group are experiencing many milestones for the first time, including moving away from home, attending colleges, starting a new job, developing new relationships, assuming new social roles, etc.—all of which are conducive to personality change. Although college‐attending samples have constituted much of psychological research (Henry, 2008), existing studies often rely on few assessment points, showing a high degree of consistency between the beginning and end of college years (e.g., De Fruyt et al., 2006; Robins et al., 2001).

To improve on this body of evidence, we included four assessment points over a short, yet important, duration of 1.5 years from the first to the third year of college. Instead of relying on sampling of college students merely for convenience, the current research explicitly investigates this college‐attending population and a process that is salient in their age group: the development of personality traits. The shorter time scales of roughly five‐month intervals are appropriate for this age group, for whom environmental and situational changes are happening rapidly, and which may prompt much more dynamic features of personal development than previously suggested by less sensitive longitudinal designs. Further, although the cumulative continuity principle posits an overall increase in rank‐order stability of personality traits across the lifespan (Roberts et al., 2008), this particular age group is also engaging in extensive identity exploration, which may temporarily result in a decrease in stability (Arnett, 2006). Our longitudinal design is well‐equipped to investigate this temporary pattern.

### 1.5 Current study

In aggregate, the current study examined both self‐ and other‐reports of personality traits in a four‐wave multiple‐informant dataset, which sensitively captures change over short, yet important, intervals during college years. Instead of restricting dyadic types, participants were free to elect their informants, often multiple, to be included in the dataset. We included the five personality domains and their 10 specific personality aspects. We assessed four types of change in personality traits and the pattern of correlations between self‐ and other‐reports. We tested four main hypotheses: (H1) Positive mean‐level change (reversed for Neuroticism); (H2) Decreasing rank‐order stability due to identity exploration; (H3) Significant individual deviation from normative overall change; (H4) Majority of ipsative profile change in elevation and scatter, not shape. In addition, we explored an exploratory research question: (Q1) Correlations and differences in change trajectories between self‐ and other‐reports. The research was preregistered prior to the first author's gaining access to the data.^1^ Further analyses were included in the preregistration and conducted on variables relating to identity development. They were excluded from the current paper to maintain the focus on personality traits, but the results are available on the linked OSF repository.

## 2 METHODS

### 2.1 Participants

Participants were recruited during the spring semester of their first year in a large Mid‐Western public university in the United States. The first wave included 259 participants. Due to attrition, the second included 196 participants, the third included 191, and the fourth wave included 150. The sample was predominantly White (75.8%), 13.5% Asian, 3.9% Mixed, 2.3% non‐European White, 2.3% Latino/a, and 1.9% Black. Slightly over half (57.4%) of participants identified as female. Consistent with the sampling population, the mean age at baseline was 18.79 years old (SD = 0.83), ranging from 18 to 25 years old. 93.1% of participants were born in the United States, and those who were not had spent a median of 5 years living in the US. Each participant nominated at least one close other, with a total of 398 other informants. The average age of informants was 29.29 (SD = 15.35) with a wide range from 18 to 73 years old. 99 informants (24.9%) were male and 298 (74.9%) were female. The majority were either a friend (50.5%) or family member (37.4%), with the remainder being either roommates, significant others, or teachers/mentors. Most informants (90.5%) indicated knowing the targets either “quite well” or “extremely well”, with only two informants indicating knowing the targets “not well at all.”

### 2.2 Procedures

The dataset was retrieved from the Personality Projects and the Development of Virtue archive (DeYoung et al., 2015). For recruitment, an email was sent to all students enrolled in a first‐year undergraduate experience course and those in the research experience pool of the university. The study coordinator followed up with participants about subsequent waves of data collection. These primary participants were instructed to nominate up to three people over the age of 18 who know them well, which resulted in an informant pool that consisted mostly of close others. Participants completed the measures on computers in the lab using Qualtrics software. Each session took one to two hours, including measures outside of the current study. Participants were paid $25 at the first wave of data collection, $30 at the second wave, $35 at the third wave, and $40 at the fourth and final wave. Data were collected at roughly six‐month intervals beginning in the spring of their first year of college and ending in the fall of the third year. Overall, data collection spanned roughly one and a half years.

### 2.3 Personality traits

The Big Five Aspect Scales (BFAS; DeYoung et al., 2007) measure five personality trait domains and their 10 aspects: Agreeableness (Compassion and Politeness), Conscientiousness (Industriousness and Orderliness), Extraversion (Assertiveness and Enthusiasm), Neuroticism (Volatility and Withdrawal), and Openness/Intellect (Intellect and Openness). Each aspect was measured by 10 items, some reverse‐scored, on a Likert scale ranging from (1) strongly disagree to (5) strongly agree. Each domain was measured by the 20 items that made up its two aspects.

### 2.4 Data preparation and analytic plan

Because multiple informants were often available for each participant, one informant was selected randomly from a pool of informants for which at least two assessment points were collected. As a result, although each participant could select multiple informants within and between waves, our analytic plan ensured that there is only one (randomly selected) informant to provide the longitudinal data. As is the case for most longitudinal research, the current study encountered missingness due to attrition. Comparisons between groups that showed some missingness versus no missingness are presented in Appendix A, Table A1. Overall, male participants were more likely to have some missingness compared to female participants and transfer students are more likely to have some missingness compared to non‐transfer students (*p* < .001). The group with some missingness also showed slightly higher age (*p* < .001) and year in school (*p* = .002) at baseline compared to those with completed data, although this difference was small in magnitude. Most of our analyses, such as mixed‐effects models, are robust to missingness in longitudinal analyses. Nonetheless, we used the *mitml* R package (Grund et al., 2016) for multiple imputations to create 20 imputed datasets, on which all analyses were conducted, and results were pooled to examine robustness between original and imputed datasets (Appendix A, Tables A2 and A3).

All analyses were conducted using R software, version 4.0.2^2^ (R Core Team, 2020) with an alpha threshold of 0.01 for research questions under the null hypothesis significance testing framework (excluding H4 of ipsative change). For research question H1 (mean‐level change), we used linear and quadratic mixed models in packages *lme4* (Bates et al., 2015) and *lmerTest* (Kuznetsova et al., 2017). The measured outcomes are 10 personality aspects and five personality domains with random intercepts and slopes. Linear and quadratic models were compared for each variable. For research question H2 (rank‐order change), we computed bivariate correlations between different waves and performed Fisher's *z*‐transformation using the *psych* package (Revelle, 2020). *Z*‐difference scores were computed to test the significance of the difference between correlations between the 1st and 2nd waves versus correlations between the 3rd and 4th waves. For research question H3 (individual differences in change), we conducted model comparison between a model with random slopes and one without random slopes, both of which have random intercepts.

For research question H4 (ipsative change), we computed three profile indices of squared Euclidean distances. In the case of two variables using the Cartesian coordinates, we can visualize this as the squared length of the line that connects two points. For an *n*‐dimensional Euclidean space, the equation is: $d^{2}={\left(p_{1}-q_{1}\right)}^{2}+\ldots +{\left(p_{n}-q_{n}\right)}^{2.}$

For our five personality domains, this becomes:

(1) $$\begin{array}{c} d_{\mathrm{ij}}^{2}={\left(AG_{i}-AG_{j}\right)}^{2}+{\left(CO_{i}-CO_{j}\right)}^{2}+{\left(EX_{i}-EX_{j}\right)}^{2}\\ \;+{\left(NE_{i}-NE_{j}\right)}^{2}+{\left(OP_{i}-OP_{j}\right)}^{2} \end{array}$$

where AG = agreeableness, CO = conscientiousness, EX = extraversion, NE = neuroticism, OP = openness, and $i\in \left\{1,2,3\right\}\;$ and $j\in \left\{2,3,4\right\}$ for the four assessment waves. Each computed $d_{\mathrm{ij}}^{2}$ thus corresponds to the squared Euclidean distance between personality profiles between two different waves. We computed these distances using raw scores (D2), deviation scores (D′2), and standardized scores (D″2) of personality domains and aspects to separate the effects of profile elevation, scatter, and shape (Cronbach & Gleser, 1953). These levels are sequential, such that the distance of raw scores also include effects of scatter and shape, whereas the standardized scores are no longer influenced by the elevation or scatter of the profiles. As a result, the effects found in profile shapes would reflect only the configuration among the personality variables, unaffected by the mean level and variability.

We used simulation to determine presence of changes based on these computed Euclidean distances. In particular, 99.9% confidence interval cut‐offs were determined by simulated trait scores for 50,000 participants based on (1) observed means, variances, and covariances, (2) no change or perfect between‐wave correlation, and (3) observed Cronbach's alpha as attenuation factors. Because we could control the true change pattern to be null for the simulated data, if the computed Euclidean distances fall outside of the simulated interval, they would be considered greater than chance‐level, and we would consider that individual to have shown profile changes. Simulations were modeled after previous literature (De Fruyt et al., 2006; Robins et al., 2001) and conducted using packages *simsem* (Pornprasertmanit et al., 2020) and *simstudy* (Goldfeld, 2020).

For exploratory research question Q1 (differences in change trajectories between self‐ and other‐reports) we extracted the predicted individual slopes from linear models fitted in H1 and examined their correlation patterns. All reported analyses were preregistered unless explicitly noted.

In addition to analyzing self and other reports separately, we also conducted non‐preregistered analyses in which we created latent factors with indicators from both reports to offset informant‐specific response bias. Latent growth and latent stability models were used to examine linear change as well as rank‐order change in personality traits using the *lavaan* package (Rosseel, 2012). Due to the high number of item indicators, four random parcels were created for each latent factor to better facilitate model convergence (Matsunaga, 2008), with two parcels per informant which remained the same across waves. Latent means for slopes were estimated to examine change in variables over time, with loadings of 0, 6, 13, 19 which corresponds to time from baseline measured in months. Stability models regressed latent variables at each wave on the same variable the prior wave. Residual covariances were included among similar parcels across waves. Full information maximum likelihood was used to account for missing data.

## 3 RESULTS

### 3.1 Mean‐level change

There was no evidence for linear change in personality domains or aspects in our sample. Changes in all personality variables did not reach statistical significance at our alpha threshold of 0.01. This lack of mean‐level change was replicated in all linear models in other‐reports.

**FIGURE 1:**
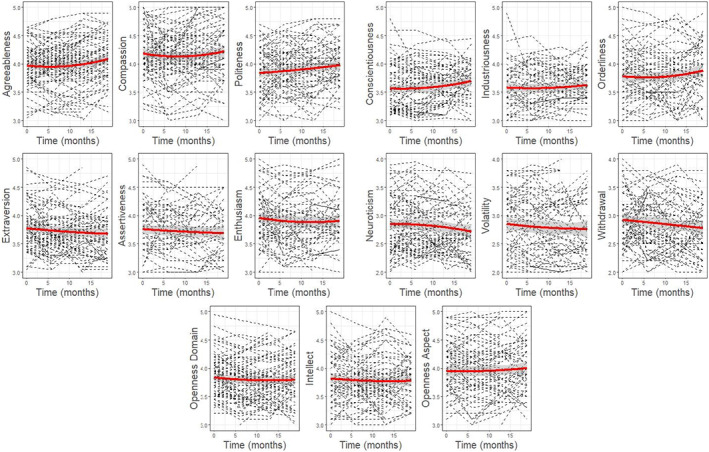
Linear and quadratic trends for the self‐reported personality traits. Time is shown in months from baseline assessments. A random sample of 100 participants was selected for individual slope lines.

In order to examine the presence of quadratic trends in change, linear and quadratic models were fitted for each variable. If model comparison indicated that the quadratic model fit the data significantly better than the linear model, we conclude that there was evidence of a curvilinear change pattern. Such a pattern was found only for Extraversion (*χ* ^2^ = 8.49, *p* = .004) and its aspect Assertiveness (*χ* ^2^ = 6.82, *p* = .009). For these variables, the quadratic pattern was a slight U‐shape, signifying that trait scores initially decreased before the eventual increase (Figure 1). This effect was very modest, with *B* estimates of 0.0006 for both Extraversion (*t*_[394.05]_ = 2.93, *p* = .004) and Assertiveness (*t*_[394.30]_ = 2.62, *p* = .009) on a five‐point Likert scale.

**FIGURE 2:**
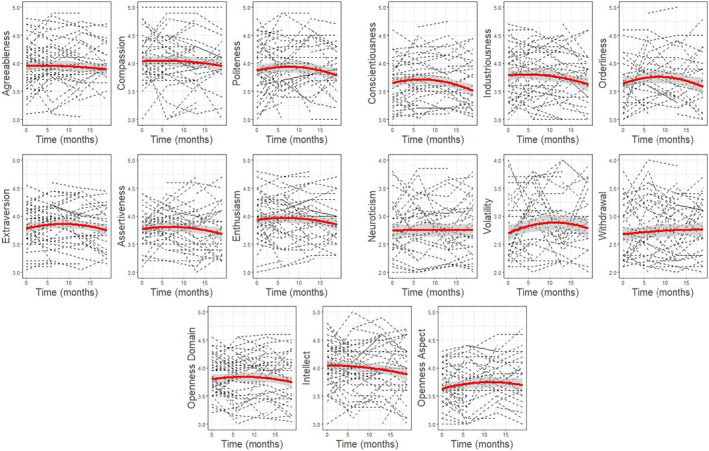
Linear and quadratic trends for the other‐reported personality traits. Time is shown in months from baseline assessments. A random sample of 100 participants was selected for individual slope lines.

For other‐reports, the quadratic model performed better for only the aspect Orderliness (*χ* ^2^ = 8.03, *p* = .005), but not any Big Five domains. Contrary to self‐reports, the quadratic pattern was an inverted U‐shape, with scores initially increasing before the eventual decrease (Figure 2). The effect was also modest in magnitude (*B* = −0.001, *t*_[252.19]_ = −2.86, *p* = .005). A summary of both self‐ and other‐results are presented in Table 1.

**TABLE 1:** Linear mean‐level change for personality traits for self‐ and other‐reports

		Self‐reports			Other‐reports		
Domains	Aspects	Slope	95% CI	*p*	Slope	95% CI	*p*
Agreeableness		0.002	0.000 to 0.005	.086	−0.002	−0.006 to 0.002	.255
	Compassion	0.002	−0.002 to 0.005	.355	−0.001	−0.005 to 0.004	.808
	Politeness	0.003	−0.000 to 0.006	.073	−0.004	−0.009 to 0.001	.098
Conscientiousness		0.002	−0.001 to 0.005	.123	−0.002	−0.006 to 0.001	.213
	Industriousness	0.001	−0.003 to 0.004	.754	−0.003	−0.007 to 0.002	.232
	Orderliness	0.004	0.001 to 0.008	.025	−0.002	−0.007 to 0.003	.378
Extraversion		−0.001	−0.004 to 0.002	.396	0.001	−0.002 to 0.005	.443
	Assertiveness	−0.001	−0.005 to 0.002	.504	0.001	−0.003 to 0.005	.576
	Enthusiasm	−0.001	−0.005 to 0.002	.483	0.001	−0.003 to 0.006	.567
Neuroticism		−0.001	−0.005 to 0.002	.489	0.004	−0.000 to 0.009	.053
	Volatility	−0.001	−0.005 to 0.003	.649	0.006	−0.000 to 0.011	.055
	Withdrawal	−0.002	−0.005 to 0.002	.418	0.003	−0.001 to 0.008	.169
Openness		0.001	−0.002 to 0.003	.558	−0.003	−0.006 to 0.000	.078
	Openness	0.001	−0.001 to 0.004	.333	−0.001	−0.005 to 0.002	.466
	Intellect	−0.000	−0.003 to 0.003	.961	−0.005	−0.009 to −0.001	.027

*Note*: Slope estimates with 95% confidence intervals and associated *p*‐values are shown for five domains and their ten aspects (BFAS, DeYoung et al., 2007). None of the reported estimates was significant at the 0.01 alpha level.

### 3.2 Rank‐order change

**FIGURE 3:**
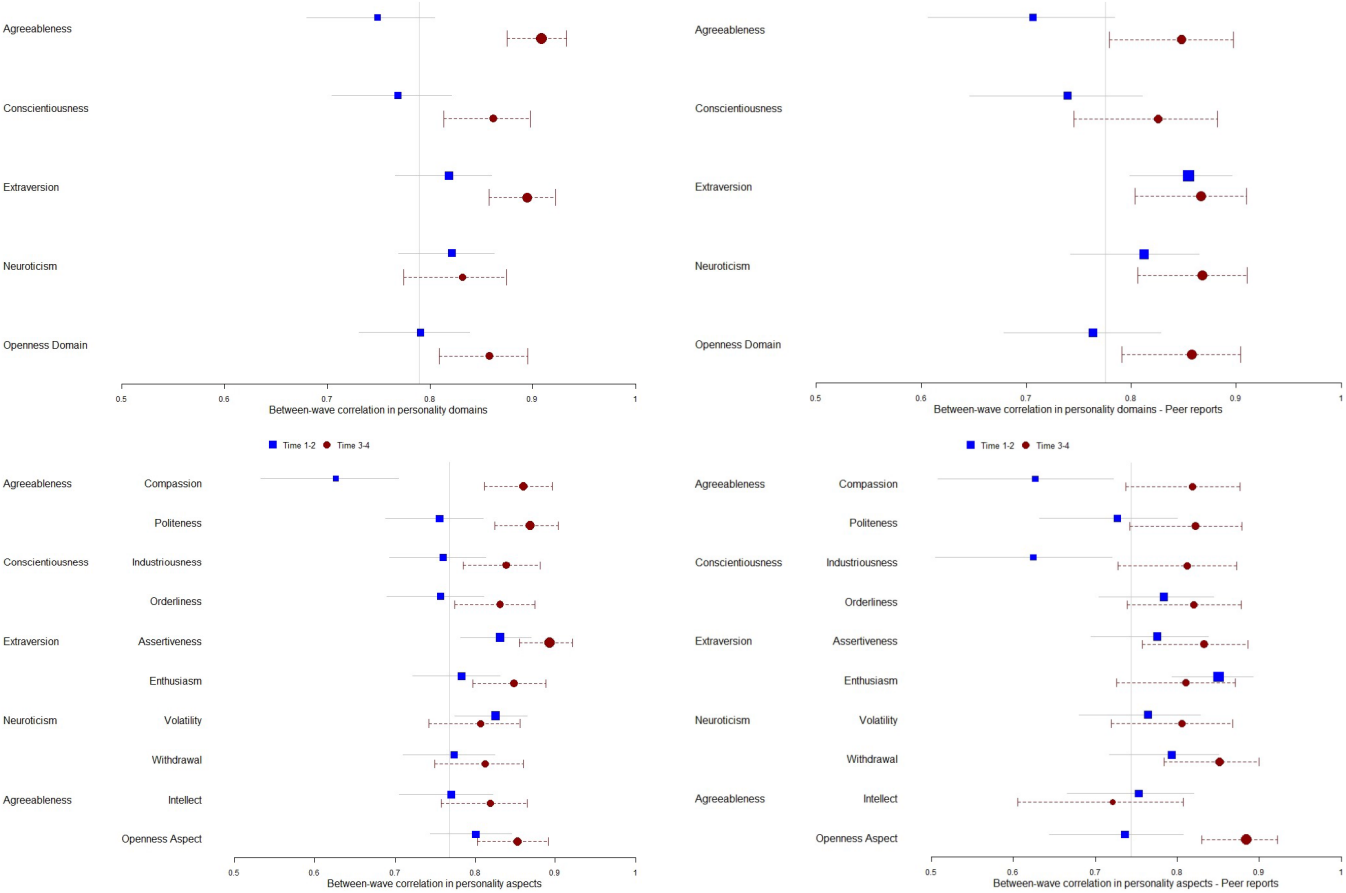
Forest plots comparing correlations between the first two waves and the last two waves for personality domains and aspects for self‐reports (left) and other‐reports (right).

**FIGURE 4:**
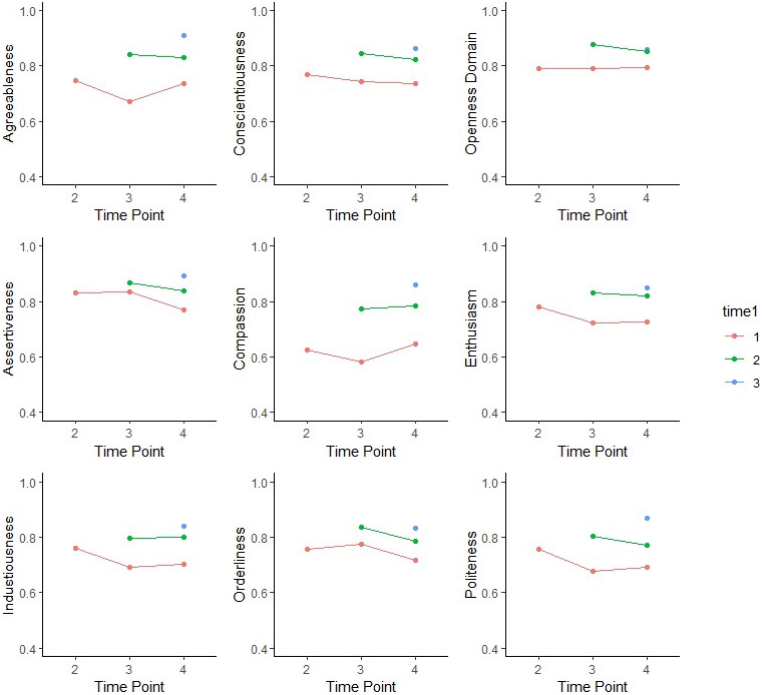
Temporal decay curves for self‐reported (1) domains Agreeableness, Conscientiousness, and Openness; (2) aspects Assertiveness, Compassion, Enthusiasm, Industriousness, Orderliness, and Politeness. Graphs of the nine personality traits showed significant increases in rank‐ordering over time: correlations between the last two waves are higher than correlations between the initial two waves. In addition, the step ladder shapes confirmed the longitudinal patterns in which assessments closer together are more strongly correlated than those further apart.

All personality variables were strongly and significantly correlated across waves, which was evidence of rank‐order stability. This is expected, due to the short duration of the study and the relative stability often found for these variables. What the current study is interested in, however, is whether or not this correlation decreases or increases over time. Correlation coefficients *r* was transformed to *z*‐scores with Fisher's *z* transformation and compared between different intervals. For most of the personality variables, correlations between the first two waves were significantly weaker than correlations between the last two, with *z* difference‐scores ranging from 2.27 (*p* = .004) for Extraversion to 5.01 (*p* < .001) for Agreeableness. Notably, this effect was not found for the domains Neuroticism (*z* = −0.29, *p* = .384) and Openness/Intellect (*z* = −1.94, *p* = .026) or any of their four aspects. Forest plots comparing correlations between the first two waves and the last two waves are presented in Figure 3. These patterns are further demonstrated through temporal decay curves (Figure 4), which plotted all between‐wave correlations among the four available waves. For variables with significant increase in rank‐order stability over time, there is a step‐ladder pattern in which subsequent waves correlated lowest with the first wave, and correlation generally increased until the last two waves.

**FIGURE 5:**
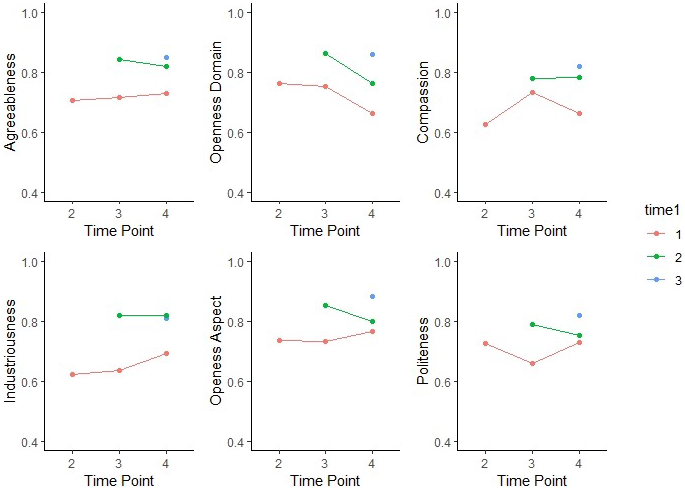
Temporal decay curves for other‐reported personality traits. Graphs of the six personality traits showed significant increases in rank‐ordering over time: correlations between the last two waves are higher than correlations between the initial two waves. In addition, the step ladder shapes confirmed the longitudinal patterns in which assessments closer together are more strongly correlated than those further apart.

Similar to self‐reports, all other‐reported personality variables were strongly and significantly correlated across waves. However, increasing stability was found only for Agreeableness (*z* = 2.66, *p* = .004) and its Compassion aspect (*z* = 3.00, *p* = .001), in which correlations between the first two waves were significantly weaker than correlations between the last two. Although the aspects Industriousness (*z* = 2.87, *p* = .002) and Openness (*z* = 3.25, *p* = .001) showed similar stability increase, their corresponding domains did not. These patterns are demonstrated through temporal decay curves (Figure 5).

### 3.3 Individual differences in change

In order to determine the existence of individual differences in change, two linear models were fitted for each variable, one with both random intercepts and random slopes, and one with only random intercepts and no random slopes. If the first model performed better than the second model, we may conclude that the random slope terms were adding significant information; that is, there are significant individual differences in change. This hypothesis was confirmed through model comparison for most personality variables, signifying that participants not only vary in their scores at baseline but also their change trajectories over the 2.5‐year period. The exceptions to this pattern were the aspect Withdrawal (*χ* ^2^ = 4.35, *p* = .114) and the domain Openness/Intellect (*χ* ^2^ = 3.21, *p* = .201) with its two aspects of Intellect (*χ* ^2^ = 4.06, *p* = .131) and Openness (*χ* ^2^ = 0.95, *p* = .623). Other‐reports showed similar patterns for most personality domains—scores varied not only at baseline but also in their change trajectories, except for the two domains Agreeableness (*χ* ^2^ = 1.58, *p* = .455) and Conscientiousness (*χ* ^2^ = 2.43, *p* = .297). Notably, the only other‐reported personality aspect that demonstrated significant individual slopes was Enthusiasm (*χ* ^2^ = 25.28, *p* < .001). These patterns are shown in Figures 1 and 2, in which a random subset of 100 participants were selected to plot individual slope lines against the aggregated estimated slope at the sample level.

### 3.4 Profile change

To examine ipsative profile change, squared Euclidean distances were computed to represent the distance between personality profiles between different timepoints (Cronbach & Gleser, 1953). We used three indices: the raw scores showed effects of all elevation, scatter, and shape of profiles; the deviation scores were not affected by elevation; and the standardized scores showed only an effect of shape. This is the core of our research question: whether the shape or configuration of personality profiles changes during college years.

**FIGURE 6:**
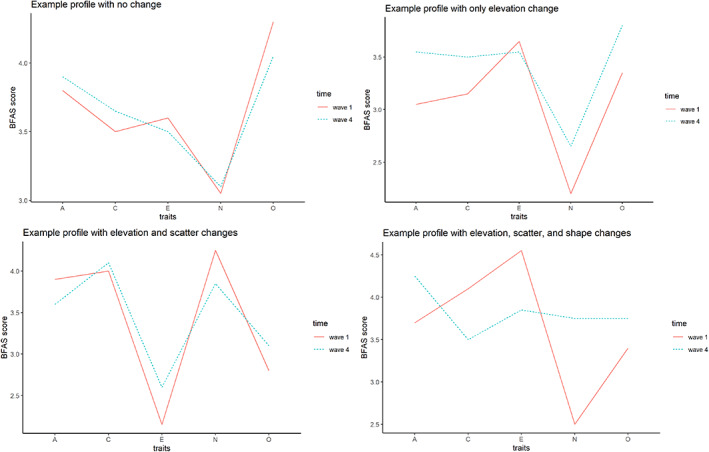
Case examples to illustrate ipsative profile changes. Four random participants were selected who showed no profile change (top left), showed only change in elevation (top right), showed changes in elevation and scatter (bottom left) and showed changes in all elevation, scatter, and shape (bottom right). Profile changes were examined between the first and last waves.

The middle 99.9% of the simulated null distribution was extracted; if our results fell outside of this distribution, they were considered to be greater than chance‐level. For profiles of the five personality domains, 50%–51% of participants showed notable profile change using raw and deviation scores (51%–54% for other‐reports). The percentage dropped to 35% (39% for other‐reports) when using standardized scores, signifying that much of the profile change was in elevation and scatter, not in configural shape. In contrast, for profiles of the 10 personality aspects, change did not differ depending on type of index, and about half of participants (50%–54% for self‐reports and 48%–51% for other‐reports) showed notable profile change with either raw, deviation, or standardized scores. To further illustrate the meaning of these different profile change types, Figure 6 provides the profile plots for four random individuals from our sample who (1) did not have profile change, (2) had changes in elevation but not scatter or shape, (3) had changes in elevation and scatter, but not shape, and (4) had changes in all elevation, scatter, and shape.

### 3.5 Correlated change across traits and across informants

#### 3.5.1 Across traits

Although we did not find significant sample‐level change in mean scores for most variables, due to the significant individual differences, we wanted to take a closer look at individual‐level patterns of correlated change. There were a few intriguing correlated changes across traits, particularly with self‐perceived change in Intellect, which showed a negative correlation with other‐perceived change in Conscientiousness (*r* = −.19) and positive correlation with other‐perceived change in Neuroticism (*r* = .17). However, none of these correlations was significant at the .01 alpha level and thus additional research with larger samples might be needed to confirm these patterns (Table B2).

#### 3.5.2 Across informants

Although there were strong positive correlations between self‐ and other‐reports at baseline (all *p*s < .01; Table B1), notably, none of the 17 change variables showed significant correlations between self‐ and other‐reports (*p* > .05), indicating a lack of evidence for convergence in self‐ and other‐perception of change (Table B2; for references, Table B3 provided internal consistencies of each scale at each wave and for each informant). Given this unexpected pattern of results, we performed exploratory non‐preregistered analyses to test informant type as a moderator of self‐other agreement. Informant type was coded as either (1) family members or (2) friends and roommates. A regression model was fitted for each of the 10 aspects and 5 domains, with other‐reported slope, informant type, and their interaction predicting self‐reported slope. The interaction term was not significant for any model at an alpha of 0.01.

## 4 DISCUSSION

The current research benefited from the rich longitudinal multi‐informant dataset that allowed us to examine both self‐ and other‐perceptions of change through four assessment waves within a relatively short period of time during college years – a critical period of self‐exploration in emerging adulthood. Through both sample‐level and individual‐level approaches, we examined a holistic picture of personality change and demonstrated how change may be differentially perceived through different conceptualizations, including mean‐level patterns, rank‐ordering, individual differences, and ipsative profiles over time. In addition, our individual analyses explored not only individual traits separately, but also the pattern of configural change as well as correlated change between self‐ and other‐reports. This comprehensive approach further elucidates individual‐level patterns that may have been hidden from a purely population‐level, univariate examination as commonly seen in the literature. In short, despite little to no evidence of change in a mean‐level approach, each successive analysis of change demonstrated a more dynamic picture. Given the numerous examined variables and associated tests and potential researcher's degree of freedom, the study was preregistered prior to the first author gaining access to the existing data. The preregistration process was intended to be comprehensive such that we pre‐specified confirmatory hypotheses, exploratory questions, analysis plan, as well as a full R script file designed and tested on simulated data.

### 4.1 Self‐reports

Our result pattern showed differential support for our hypotheses. In particular, we did not find any mean‐level change in personality traits. Further, pooled results from multiple imputations (Appendix A) did not show any significant linear mean‐level effects. A few personality variables suggested a quadratic U‐shaped, rather than linear, trajectory over the 2.5‐year period. Although the effects were modest, this suggests an eventful time period for change and further confirms the importance of including multiple time points over a relatively short duration to sensitively capture the shape or functional form of change.

Compared to mean‐level change, rank‐order stability in personality traits showed more changes, with an overall increase in sample‐level stability across four waves. That is, correlations between the later waves were stronger than correlations between the earlier ones. This provided support for the cumulative continuity principle of increasing rank‐order stability across the lifespan. In addition, it disconfirmed our hypothesis that this age group in particular would experience a temporary decrease in stability due to the salient identity exploration process. In contrast to mean‐level change, multiple imputations showed significant increase in rank‐order stability for all but one variable (Volatility) even when results of original data with missingness did not (Appendix A, Table A3). Given the nature of the two analyses, this discrepancy is expected. Whereas the mixed model used to analyze mean‐level change was well equipped to accommodate missingness, rank‐order analyses included bivariate correlations that did not allow for missingness. Thus, multiple imputations produced notably different results.

Mean‐level and rank‐order changes, although much different in nature, both approach changes from a sample‐level perspective. At any stage in the lifespan, but perhaps most prominently during emerging adulthood (Arnett, 2000), we should expect individual variations in the overall change trajectories. This pattern was confirmed for most personality traits. That is, there were significant deviations from the normative overall pattern of change, meaning that individuals have varying trajectories over the 1.5‐year period. This significant variability in change trajectories illustrates the varied experiences of emerging adults during their college years. These varied influences may thus lead to varied developmental processes. For each personality trait, some participants showed mean‐level increases, others showed decreases, whereas some showed no change at all. Many are immersed in identity exploration and with diverging interests, acquaintances, and environments. Thus, the aggregate mean‐level results demonstrated a limited picture of the actual patterns of change.

Our last approach to change, and the least common approach in the literature, is configural profile changes. Using Euclidean distances and simulated trait scores, we replicated existing findings in the literature (De Fruyt et al., 2006; Robins et al., 2001): configural changes were mostly due to profile elevation (or level) and scatter. Notably, however, a non‐negligible proportion of participants (35%) still showed shape changes in personality domain profiles, compared to the 10% and 17% previously reported across two assessment time‐points with children, adolescents, and undergraduate students (De Fruyt et al., 2006; Robins et al., 2001). This indicated that there was an overall change in the configuration of personality profiles and relative relationship among different traits, beyond a surface‐level change in overall mean scores or variability across traits.

Personality profiles using the 10 aspects showed much more configural change compared to profiles with the five domains. It follows logically that the configuration with more components would be more sensitive to change. Yet, it was unexpected that the pattern remained stable for aspect profiles regardless of the raw, deviation, or standardized indices used, averaging at about half of participants (50%–54%) showing notable profile changes in elevation, scatter, and shape. This further illustrated the benefits of including more fine‐grained traits in the analysis of personality change. In particular, with the aspect level, our computed profile indices not only capture the relations between orthogonal domains, but also the interactions between aspects that belong to the same overarching domain. Thus, we were able to examine the change processes more sensitively and, if change were to occur, we would have more power to examine the source of that change. For instance, changes in the shape of the five domains would not capture the differential change pattern between, say, Assertiveness and Enthusiasm, because these aspects are combined to form the Extraversion domain. Nonetheless, they provide substantively different information about an individual's personality make‐up. It is evident from the overall results that sample‐level approaches might not adequately demonstrate the change patterns at play, particularly due to the significant between‐person variations in individual change trajectories.

### 4.2 Self‐reports versus other‐reports

Our multi‐informant dataset allowed for the comparison between self‐reported and other‐reported trajectories over the 1.5‐year period. Bivariate correlations between self‐ and other‐reports at baseline and in slopes are reported in Appendix B. Notably, participants were free to nominate their other informants of choice, and thus we had informants ranging from classmates, friends, romantic partners, to family members. Although baseline correlations suggested high self‐informant agreement (Table B1), we were interested in whether, in aggregate, changes are perceived similarly from the outside (Table B2). That is, can close acquaintances tell that you have changed?

Overall, much of the mean‐level change pattern, or rather the lack thereof, was replicated in other‐reports. Similar to self‐reports, others did not report significant linear change. The quadratic trend prevalent in self‐reports was not found for much of other‐reported personality traits. Interestingly, for the other‐reported personality variable with quadratic change (Orderliness), the patterns were inverted: whereas self‐reports showed a U‐shape for most traits, other‐reports showed an inverted U‐shape, with scores initially increasing before the eventual decrease. Effects were once again very modest.

Similar to self‐reports, others also reported significant rank‐order stability, which showed an increase over time such that correlations between the first two waves were lower than correlations between the last two, although this pattern was found in relatively fewer traits in other‐reports. In addition, ipsative analyses estimated a similar proportion (about half) that showed changes in elevation and scatter of personality domain profiles, with a drop to about a third showing change in profile shape. As expected, this proportion is higher for profiles with many components (10 personality aspects) than for those with few components (five personality domains).

The most striking deviation between self‐ and other‐reports was shown through correlation patterns between individual extracted slopes from linear mixed models, in which none of the 17 examined variables showed significant correlated change. That is, for our age group in the current short time span, any self‐reported longitudinal pattern of change in personality traits was not similarly perceived by close others. It is important to note that our study covers a relatively short time duration of one and a half year, and thus this might suggest either (1) a discrepancy between self‐ and other‐perceived change or (2) a time lag in this perception, in which close others failed to perceive change in personality traits immediately after participants reported these changes. It may simply take additional time for people to update their mental models of those they are close to, as they have less opportunity to observe the target person's behavior than the target has to observe their own behavior. Notably, the current research focused on close others and the data are not suitable to address the potential impact of level of acquaintanceship because we do not have a wide range of familiarity. Almost all informants indicated knowing the participants well, and informant type (family members versus friends) did not moderate self‐other agreement. As a result, future studies on a range of informants might produce divergent findings and additional insight on self‐other agreement on change.

**FIGURE B1:**
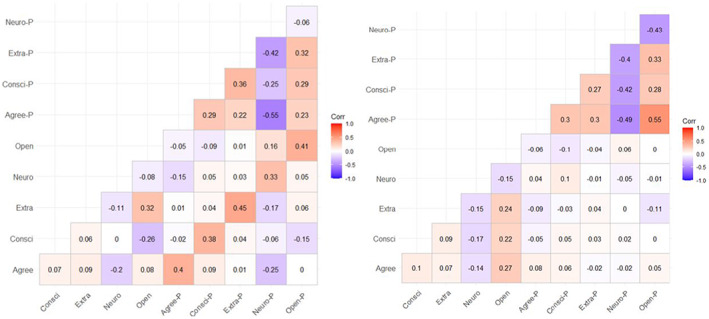
Multi‐trait multi‐method correlation matrices between self and other reports at baseline and as linear slopes. Left: baseline correlations; Right: slope correlations. ‐P indicates peer‐ or other‐reports. Agree: Agreeableness; Consci: Conscientiousness; Extra: Extraversion; Neuro: Neuroticism; Open: Openness/Intellect domain.

To mitigate the potential rater bias suggested by the multi‐method multi‐trait matrices (Figure B1), we created latent variables that combined self‐ and other‐ratings and fit growth and stability models. These results did not differ meaningfully from the separate self‐ and other‐analyses using observed scale scores. However, it is important to note that our structural equation models were a highly exploratory supplement to the main analyses, with only moderate fit and several convergence problems. Additional details are provided in Appendix C, and they should be interpreted with much caution.

### 4.3 Limitations and future directions

Although the research program greatly benefited from a rich longitudinal multi‐informant dataset, our sample size was moderate, with 259 participants at baseline, and was further reduced over time to 150 participants at the fourth and final wave. The full study procedure was extensive, for which participants were required to attend in‐person sessions spanning 1–2 hours, and attrition was inevitable given the longitudinal design. In addition, we retained informant data for only 176 of the total 259 participants, because some participants always nominated different informants at different time points and were thus ineligible for our longitudinal analyses.

The modest sample size means that our analyses, particularly our structural equation models, were underpowered to detect significant effects. In addition, our alpha threshold was preregistered at 0.05 due to our modest sample size. Nonetheless, given the extent of multiple testing, we have formally changed this threshold to 0.01 instead of adding notes of caution for our few “borderline” *p*‐values between .05 and .01. The current paper included multiple families of tests, each with its own clustering of trait and informant variables. As a result, alpha adjustments are difficult to enforce across analyses to achieve the same family‐wise error rate. For future research, we recommend instead to plan for separate adjustments a priori if researchers investigate multiple types of change, each with its own analytic approach.

Further, the sample was limited in that it included mostly White college students in a mid‐western university in the United States and thus might not be generalizable to other demographic groups within emerging adulthood. Although the short time span was designed to sensitively capture change patterns during this turbulent period of identity development, we suspect it might be helpful to further increase assessment frequency for even shorter time intervals. Because the exact timeline of these developmental processes is unclear, and because of the high degree of individual differences in these trajectories, it is entirely possible that different time spans and assessment frequencies might produce different results.

It is important to note that our hypotheses were not atheoretical, as the hypothesized directions of change were based on both the general conceptualization of personality stability and established principles in relation to lifespan personality development. However, we did not hypothesize any mechanisms or correlates of change. Although this was by design, as the research was intended for theory‐guided observational purposes, the findings of individual differences naturally introduced follow‐up questions to examine the potential sources or predictors of change. Thus, our research program will prove to be a useful foundation for future research to explicitly examine these factors.

## 5 CONCLUSIONS

The current study provided a comprehensive examination of different kinds of changes in personality traits in college‐attending emerging adults over one and a half years. The four‐wave, multi‐informant dataset allowed analyses of both self‐ and other‐ratings, as well as a longitudinal comparison between the different informants. Overall, we found differential support for our hypotheses such that, although not much mean‐level change was observed during the short study period, participants showed significant individual differences in change trajectories, and rank‐ordering and ipsative profiles showed much more changes than mean levels. Informant‐reports from close others of various relationship types demonstrated largely similar patterns: little to no mean‐level change, significant increase in rank‐ordering, and about half of participants showing ipsative change mostly in elevation and scatter rather than profile shape. The significant variability in change trajectories in this sample converged with previous research on lifespan trajectories and was consistent with the exploration and life‐change processes that are salient to this age group.

Notably, similar to past research in emerging and middle adulthood but in contrast to longer‐term research programs on older adults, there was no evidence of correlated change between self‐ and other‐breports, suggesting that close others do not share our perception of personality change, at least not in the current age group and timespan. By examining different conceptualizations of change, we provided what we hope will be a useful observational foundation for future research to examine the source of variability in change trajectories, as well as potential moderators of the relation between our own and our close others' perception of personality change.

## AUTHOR CONTRIBUTIONS

All authors were involved in the data analytic plan and manuscript revision. Phuong Linh L. Nguyen was responsible for conceptualization, data analysis, and drafting of the paper. Moin Syed and Colin G. DeYoung were responsible for data collection and revising/editing the paper.

## ETHICS STATEMENT

The authors had no conflict of interest. This study was approved by the Institutional Review Board of the University of Minnesota.

## Endnotes

^1^
Preregistration document: https://osf.io/hf9sv.

^2^
Full analysis script and data (without demographic information) are included in the OSF repository: https://osf.io/fyaeg.

## APPENDIX A MISSINGNESS AND MULTIPLE IMPUTATION

**TABLE A1:** Comparisons of Gender, Age, Year in school, and Transfer status between participants with completed data versus those with missing data

Demographic variable	Not missing	Missing	*p*‐value
Gender			
Male (%)	198 (67.8)	94 (32.2)	<.001
Female (%)	593 (80.1)	147 (19.9)	
Transfer student			
No (%)	763 (78.2)	213 (21.8)	<.001
Yes (%)	31 (51.7)	29 (48.3)	
Age			
Mean (SD)	18.7 (0.7)	19.0 (1.1)	<.001
Year in school			
Mean (SD)	1.1 (0.4)	1.2 (0.7)	.002

*Note*: Missingness in the Agreeableness variable was used to represent general missingness patterns. Results did not differ significantly when using other personality variables.

**TABLE A2:** Linear mean‐level change for personality traits for self‐ and other‐reports

		Self‐reports			Other‐reports		
Domains	Aspects	Slope	Slope MI	*p* MI	Slope	Slope MI	*p* MI
Agreeableness		0.002	0.003	.454	−0.002	−0.003	.374
	Compassion	0.002	0.002	.599	−0.001	−0.002	.688
	Politeness	0.003	0.003	.371	−0.004	−0.004	.357
Conscientiousness		0.002	0.003	.479	−0.002	−0.001	.706
	Industriousness	0.001	0.000	.981	−0.003	−0.003	.403
	Orderliness	**0.004**	**0.005**	**.156**	−0.002	−0.000	.986
Extraversion		−0.001	−0.001	.818	0.001	0.001	.712
	Assertiveness	−0.001	−0.001	.748	0.001	0.003	.419
	Enthusiasm	−0.001	−0.001	.734	0.001	−0.001	.865
Neuroticism		−0.001	0.001	.819	0.004	0.006	.114
	Volatility	−0.001	−0.000	.916	0.006	0.005	.243
	Withdrawal	−0.002	−0.002	.664	0.003	0.006	.204
Openness		0.001	0.001	.671	−0.003	−0.004	.169
	Openness	0.001	0.001	.702	−0.001	−0.004	.315
	Intellect	−0.000	0.001	.766	**−0.005**	**−0.005**	**.091**

*Note*: Slope estimates using the original data and pooled multiple imputation results (Slope MI) along with the associated *p*‐values (*p* MI) are shown for five domains and their ten aspects (BFAS, DeYoung et al., 2007). Bolded are the variables showing significant linear change using original data (self‐reported Orderliness and other‐reported Intellect) that are no longer significant.

**TABLE A3:** Rank‐order change for personality traits for self‐ and other‐reports

Domains	Aspects	Self‐reports			Other‐reports		
		*z*‐score	*z*‐score MI	*p* MI	*z*‐score	*z*‐score MI	*p* MI
Agreeableness		−5.005	−9.230	<.001	−2.661	−3.949	.001
	Compassion	−5.098	−8.844	<.001	−2.999	−4.068	<.001
	Politeness	−3.150	−6.533	<.001	**−1.742**	**−3.801**	**.001**
Conscientiousness		−2.567	−6.574	<.001	**−1.587**	**−3.129**	**.008**
	Industriousness	**‐2.037**	**‐5.867**	**<.001**	−2.870	−3.787	.001
	Orderliness	**‐1.870**	**‐5.229**	**<.001**	−0.732	−2.578	.021
Extraversion		−2.667	−6.019	<.001	−0.317	−1.909	.064
	Assertiveness	−2.263	−4.384	.001	−1.166	−2.765	.018
	Enthusiasm	**‐1.823**	**‐4.800**	**<.001**	0.915	−1.416	.129
Neuroticism		**‐0.294**	**‐4.067**	**.001**	−1.361	−2.506	.018
	Volatility	0.507	−2.800	.019	‐0.779	−2.434	.021
	Withdrawal	**−0.954**	**‐4.327**	**<.001**	−1.310	−2.488	.020
Openness		**−1.940**	**‐6.192**	**<.001**	**−1.998**	**−4.074**	**<.001**
	Openness	**−1.539**	**‐4.871**	**<.001**	−3.252	−4.603	<.001
	Intellect	**−1.206**	**‐5.666**	**<.001**	**0.497**	**−2.853**	**.005**

*Note*: *Z*‐difference scores to compare correlation between the first two waves and the last two waves, using the original data and pooled multiple imputation results (MI). Bolded are the variables across self‐ and other‐reports for which there was no significant increase in rank‐ordering in the original data but there was a significant increase in pooled multiple imputations results.

## APPENDIX B SELF AND OTHER CORRELATIONS

**TABLE B1:** Bivariate correlations between self‐ and other‐reports of personality traits at baseline (wave 1)

_Other_	^Self^														
	A	Com	Pol	C	Ind	Ord	E	Asst	Ent	N	Vol	Wdr	O	Int	Opa
*Agreeableness*	**.41** ******	.23**	.45**	.00	.04	−.03	−.01	−.14	.14	−.17*	−.27**	−.01	−.06	−.12	.01
Compassion	.35**	**.28** ******	.30**	−.03	.05	−.08	.11	−.03	.22**	−.07	−.14	.02	.01	−.01	.02
Politeness	.39**	.13	**.50** ******	.03	.02	.02	−.11	−.22**	.04	−.23**	−.35**	−.04	−.12	−.20*	.00
*Conscientiousness*	.11	.03	.14	**.37** ******	.24**	.38**	.02	−.00	.03	.02	.02	.02	−.11	−.07	−.10
Industriousness	.12	.03	.16	.32**	**.27** ******	.25**	.07	.08	.05	−.04	−.02	−.05	−.10	−.04	−.11
Orderliness	.07	.04	.08	.32**	.14	**.38** ******	−.04	−.06	.01	.06	.04	.08	−.08	−.07	−.07
*Extraversion*	.03	.18*	−.11	.04	.14	−.05	**.44** ******	.39**	.34**	.01	.13	−.14	.01	.20*	−.16
Assertiveness	−.09	.11	−.24**	.03	.10	−.03	.34**	**.43** ******	.14	.08	.19*	−.07	.07	.26**	−.13
Enthusiasm	.13	.18*	.05	.04	.13	−.05	.40**	.24**	**.44** ******	−.07	.02	−.16	−.05	.09	−.15
*Neuroticism*	−.24**	−.13	−.26**	−.05	−.08	−.01	−.18*	−.08	−.23**	**.32** ******	.31**	.26**	.13	.09	.12
Volatility	−.28**	−.14	−.32**	−.02	−.04	−.01	−.07	.04	−.17*	.30**	**.36** ******	.16	.10	.12	.05
Withdrawal	−.13	−.08	−.13	−.08	−.13	−.02	−.28**	−.22**	−.25**	.28**	.18*	**.33** ******	.13	.03	.18*
*Openness domain*	−.00	.04	−.04	−.17*	−.13	−.16	.04	.11	−.05	.06	.06	.05	**.39** ******	.31**	.33**
Intellect	.01	.04	−.01	−.03	.01	−.05	.07	.14	−.03	.03	.02	.03	.24**	**.32** ******	.08
Openness aspect	−.02	.02	−.05	−.27**	−.23**	−.22**	−.00	.04	−.04	.07	.08	.05	.42**	.19*	**.47** ******

* *p* < .05

** *p* < .01.

The bolded numbers in the diagonal represent correlations between self‐ and other‐reports of the same trait.

**TABLE B2:** Correlated change and between self and other reports of personality traits

_Other_	^Self^														
	A	Com	Pol	C	Ind	Ord	E	Asst	Ent	N	Vol	Wdr	O	Int	Opa
*Agreeableness*	**.01**	−.07	.09	−.04	−.04	−.03	−.11	.19*	.01	.05	.11	−.06	−.05	−.10	.04
Compassion	−.01	**−.09**	.08	.00	.03	−.03	−.06	−.15	.04	.01	.08	−.11	−.02	−.03	.01
Politeness	.01	−.05*	**.06**	−.07	−.10	−.01	−.11	−.15*	−.03	.07	.10	−.00	−.05	−.13	.05
*Conscientiousness*	.04	.06	.02	**.04**	.00	.05	−.03	−.07	.02	.10	.11	.07	−.10	−.19*	.04
Industriousness	.06	.02	.05	.01	**−.00**	.01	−.09	−.11	−.05	.05	.06	.02	−.00	−.08	.10
Orderliness	−.04	−.03	−.02	.04	−.00	**.06**	−.05	−.06	−.01	.08	.07	.08	−.14	−.18*	−.05
*Extraversion*	−.02	−.04	.02	.05	.02	.05	**.04**	.04	−.00	−.01	.05	−.13	−.04	−.01	−.00
Assertiveness	.05	.03	.05	.05	.08	−.00	.03	**.07**	−.03	−.03	.03	−.11	.03	.08	.01
Enthusiasm	−.05	−.06	−.00	.01	−.06	.07	.04	.02	**.02**	.03	.07	−.09	−.09	−.08	−.03
**Neuroticism**	.02	.02	.00	−.01	.02	−.03	.01	.01	.01	**−.05**	−.14	.05	.05	.17*	−.09
Volatility	.04	.05	.01	.05	.06	.03	.06	−.00	.06	−.08	**−.14**	.01	.02	.14	−.11
Withdrawal	−.00	−.01	−.00	−.09	−.05	−.10	−.03	.01	−.06	.02	−.06	**.10**	.07	.16*	−.06
*Openness domain*	−.02	−.02	−.00	−.01	−.05	.02	−.12	−.15	−.04	−.00	.05	−.08	**.01**	−.04	.06
Intellect	−.02	−.03	.01	−.06	−.05	−.04	−.10	−.11	−.05	−.03	.02	−.08	−.06	**−.05**	−.03
Openness aspect	−.01	.01	−.03	.13	.02	.17*	−.03	−.07	.02	.00	.02	−.04	.05	−.03	**.08**

* .01 < *p* < .05.

The bolded numbers in the diagonal represent correlations between self‐ and other‐reports of the same trait. The bolded words highlight the overarching 5 domains as opposed to the underlying 10 aspects. These are important to highlight for the readers.

**TABLE B3:** Internal consistency of the personality scales at each wave for each informant (self or other)

Domains	Aspects	Self‐reports				Other‐reports			
		Wave 1	Wave 2	Wave 3	Wave 4	Wave 1	Wave 2	Wave 3	Wave 4
*Agreeableness*		**0.817**	**0.834**	**0.854**	**0.873**	**0.906**	**0.926**	**0.925**	**0.909**
	Compassion	0.835	0.853	0.865	0.896	0.900	0.919	0.926	0.900
	Politeness	0.719	0.738	0.753	0.757	0.828	0.853	0.866	0.835
*Conscientiousness*		**0.841**	**0.823**	**0.842**	**0.855**	**0.889**	**0.903**	**0.904**	**0.881**
	Industriousness	0.814	0.823	0.801	0.802	0.865	0.886	0.896	0.894
	Orderliness	0.807	0.767	0.812	0.857	0.834	0.849	0.873	0.831
*Extraversion*		**0.880**	**0.880**	**0.883**	**0.879**	**0.889**	**0.905**	**0.900**	**0.891**
	Assertiveness	0.870	0.872	0.879	0.876	0.853	0.879	0.864	0.842
	Enthusiasm	0.846	0.854	0.846	0.860	0.854	0.879	0.870	0.853
*Neuroticism*		**0.904**	**0.908**	**0.899**	**0.907**	**0.891**	**0.907**	**0.922**	**0.940**
	Volatility	0.888	0.899	0.884	0.900	0.915	0.921	0.932	0.928
	Withdrawal	0.837	0.844	0.834	0.827	0.717	0.739	0.779	0.896
*Openness*		**0.826**	**0.828**	**0.824**	**0.829**	**0.856**	**0.844**	**0.878**	**0.884**
	Openness	0.808	0.826	0.806	0.825	0.791	0.820	0.819	0.815
	Intellect	0.769	0.765	0.766	0.740	0.854	0.824	0.872	0.881

Bolded are values corresponding to the overarching 5 domains as opposed to the underlying 10 aspects.

## APPENDIX C LATENT GROWTH AND STABILITY MODELS

The overall pattern of correlated change suggested that an evaluative consistency bias might be influencing our results. In particular, change patterns of socially desirable traits were positively correlated within self‐ and other‐reports; however, self‐other agreement in trait change was nonexistent, and correlation patterns were much stronger between‐trait within‐method than vice‐versa. This suggested a method effect, in which raters might be relying on their own implicit personality theories rather than actual personality evaluation. This pattern was demonstrated more clearly in the multi‐trait multi‐method correlation matrices (Figure B1) in which correlations within the same raters were effectively higher than correlations between different raters for the same traits. To mitigate this problem, we conducted non‐preregistered analyses in which we fit growth and stability models using latent variables that combined self‐ and other‐ratings.

Unfortunately, several models had convergence problems. In particular, neither growth nor stability model converged for any of the Big Five domain variables. At the aspect level, growth models showed no linear change with nonsignificant slope estimates (*p* > .05). Estimates and fit indices for these models are presented in Table C1. For stability models, path coefficients along with their standard errors were compared between intervals. If the point estimate for the coefficient between the first two waves was not included in the confidence interval for the estimate between the last two waves, we would conclude that there was a significant change in rank‐order stability. There was an increase in stability for Compassion, Industriousness, Intellect, Orderliness, and Politeness. On the other hand, there was a decrease in stability for Openness. These results are shown in Table C2. Notably, none of these results directly contradicted previous analyses in which scale scores were analyzed separately for self and other reports. Due to convergence problems, several alternative models were also fitted, including: domain‐level models with two aspects as parcels, models that included two latent method factors with loadings on all self‐ or other‐reported item parcels, and models with additional equality and positive constraints in item residual covariances. Unfortunately, none of these additional models facilitated convergence in the domain‐level models. Analysis code for these models is included in the OSF page.

**TABLE C1:** Fit indices and estimated latent slopes for growth models

	RMSEA	CFI	Slope	Standard error	*p*‐value
Assertiveness	0.105	0.891	−0.001	0.002	.483
Compassion	0.137	0.780	0.002	0.002	.115
Enthusiasm	0.103	0.880	−0.001	0.002	.612
Industriousness	0.098	0.868	0.000	0.001	.911
Intellect	0.100	0.868	−0.000	0.001	.801
Openness aspect	0.074	0.939	0.001	0.001	.625
Orderliness	0.085	0.910	0.002	0.002	.429
Politeness	0.073	0.928	−0.000	0.001	.922
Volatility	0.135	0.830	−0.001	0.002	.703
Withdrawal	0.108	0.867	−0.001	0.002	.572

**TABLE C2:** Fit indices and estimated path coefficients and confidence interval for latent stability models

	RMSEA	CFI	Wave 2~Wave 1	Wave 3~Wave 2	Wave 4~Wave 3	First to last interval
Assertiveness	0.112	0.891	0.90 [0.82, 0.98]	0.95 [0.87, 1.03]	0.96 [0.87, 1.04]	Null
Compassion	0.143	0.787	0.79 [0.65, 0.92]	0.88 [0.77, 0.99]	1.00 [0.90, 1.10]	Increased
Enthusiasm	0.107	0.885	0.86 [0.77, 0.96]	0.88 [0.79, 0.98]	0.95 [0.84, 1.06]	Null
Industriousness	0.102	0.873	0.89 [0.77, 1.02]	0.88 [0.78, 0.98]	1.03 [0.91, 1.15]	Increased
Intellect	0.105	0.872	0.85 [0.74, 0.96]	0.96 [0.84, 1.08]	0.93 [0.81, 1.06]	Null
Openness aspect	0.076	0.942	0.99 [0.88, 1.09]	0.94 [0.87, 1.02]	0.90 [0.81, 0.99]	Decreased
Orderliness	0.086	0.918	0.82 [0.72, 0.92]	1.04 [0.94, 1.14]	0.97 [0.86, 1.08]	Increased
Politeness	0.073	0.936	0.82 [0.70, 0.94]	1.04 [0.91, 1.18]	0.97 [0.86, 1.08]	Increased
Volatility	0.143	0.833	0.95 [0.86, 1.05]	0.88 [0.81, 0.95]	0.94 [0.84, 1.05]	Null
Withdrawal	0.117	0.862	0.89 [0.79, 0.99]	0.84 [0.74, 0.93]	0.92 [0.80, 1.03]	Null
